# Obesity as a Multifactorial Chronic Disease: Molecular Mechanisms, Systemic Impact, and Emerging Digital Interventions

**DOI:** 10.3390/cimb47100787

**Published:** 2025-09-23

**Authors:** Ewelina Młynarska, Kinga Bojdo, Anna Bulicz, Hanna Frankenstein, Magdalena Gąsior, Natalia Kustosik, Jacek Rysz, Beata Franczyk

**Affiliations:** 1 Department of Nephrocardiology, Medical University of Lodz, 90-419 Łódź, Poland; kinga.bojdo@stud.umed.lodz.pl (K.B.);; 2 Department of Nephrology, Hypertension and Internal Medicine, Medical University of Lodz, 90-549 Łodz, Poland

**Keywords:** AI-driven personalized nutrition, digital health technologies for obesity, childhood overweight, obesity treatment, obesity complications, hormonal regulation of obesity

## Abstract

Obesity is a multifactorial chronic disease resulting from complex genetic, molecular, environmental, and behavioral interactions. Its prevalence rises worldwide, affecting cardiovascular, metabolic, oncological, hepatic, respiratory, and skeletal health. Beyond caloric excess, genetic predisposition, epigenetic modifications, gut microbiota dysbiosis, endocrine-disrupting agents, circadian misalignment, and intergenerational and prenatal influences are critical determinants of obesity risk. Core pathophysiological mechanisms include insulin resistance, dyslipidemia, chronic low-grade inflammation, and neuroendocrine dysregulation of appetite and energy balance. These processes are linked to comorbidities such as type 2 diabetes, hypertension, atherosclerosis, fatty liver disease, sleep apnea, osteoporosis, and cancer. Advances in molecular profiling, metabolic phenotyping, and body composition analysis are refining obesity classification and enabling precise risk stratification. Current therapeutic strategies include behavioral interventions addressing stress-related mechanisms, pharmacological therapies such as GLP-1 receptor agonists, emerging gene therapy approaches, and bariatric surgery. Gut-derived hormones (leptin, ghrelin, GLP-1, PYY, CCK) are recognized as pivotal regulators of appetite and weight. Preventive strategies increasingly emphasize circadian alignment, while epigenetic inheritance and prenatal exposures such as maternal obesity or smoking highlight early-life programming in future metabolic health. Additionally, artificial intelligence-based platforms and personalized nutrition provide innovative opportunities for individualized prevention and management. This review synthesizes contemporary evidence on the biological basis, systemic consequences, preventive strategies, and evolving therapeutic modalities of obesity, affirming its recognition as a complex chronic disease requiring personalized, multidisciplinary care.

## 1. Introduction

Obesity is a chronic disease characterized by excessive body fat that negatively impacts health. Its prevalence is rising worldwide, making it a major public health concern. According to the WHO, obesity continues to be a major global health concern. In 2022, one in eight people worldwide was living with obesity. Since 1990, obesity rates among adults have more than doubled, while among adolescents, they have increased fourfold. That same year, 2.5 billion adults aged 18 and older were classified as overweight, with 890 million of them living with obesity. Overall, 43% of adults were overweight, and 16% were obese. Childhood obesity is also on the rise—in 2024, an estimated 35 million children under the age of 5 were overweight. Additionally, in 2022, over 390 million children and adolescents aged 5 to 19 were overweight, including 160 million classified as obese [1]. While obesity is mainly measured by body mass index (BMI), this method has limitations. It is often linked with other serious conditions such as type 2 diabetes (T2DM), fatty liver disease, heart disease, stroke, high blood pressure, sleep apnea, osteoarthritis, and certain cancers. Although some cases stem from hormonal disorders affecting the pituitary, thyroid, or adrenal glands, these are considered to be separate conditions that may contribute to obesity [2]. According to the new ICD-11, obesity is now recognized as a chronic and complex disease defined by excessive body fat that can impair health. It is usually multifactorial, influenced by environmental, psychosocial, and genetic factors. In some patients, a single clear cause—such as medications, certain diseases, immobilization, or genetic syndromes—can be identified. In contrast, the older ICD-10 classified obesity simply as a result of excessive caloric intake. The terminology has since evolved, with the current understanding referring to obesity as being caused by “energy imbalance,” reflecting a more nuanced perspective. Over time, the definition of obesity has significantly changed, moving from a simplistic view of overeating to a complex medical condition with diverse causes and health implications. Obesity was officially recognized as a disease by the American Association of Clinical Endocrinologists in 2012 and the American Medical Association in 2013, followed by other national and professional organizations. It fulfills key disease criteria, such as identifiable signs (e.g., elevated BMI) and underlying pathophysiological mechanisms (e.g., disrupted hormonal regulation of appetite), and is associated with serious complications and increased mortality. Diagnosis in adults is based on BMI: ≥30 kg/m^2^ is considered obese, 25–29.9 kg/m^2^ is overweight. In Southeast Asian populations, a BMI of ≥23 kg/m^2^ may indicate obesity due to differences in body composition. For children, obesity is defined as a BMI at or above the 95th percentile for age and sex. However, BMI is only an indirect measure of body fat and does not reflect individual health status. The term “obesity” alone also fails to capture related health complications, contributing to confusion and stigma among both the public and healthcare providers. This lack of clarity has made it difficult to treat obesity as a serious medical condition that requires coordinated action [3,4]. The usefulness of BMI as a diagnostic tool for obesity has been debated for decades. Although it is widely used due to its simplicity and affordability, BMI only estimates body fat based on height and weight and does not reflect actual body composition. While helpful in large-scale studies, it may not accurately assess individual health risks. Some experts argue that body fat percentage and indicators such as fat mass index or muscle mass give more precise insights, especially for predicting cardiovascular risk. The distribution of fat, particularly visceral adipose tissue (VAT), plays a key role in obesity-related complications due to its metabolic activity. However, not all individuals with obesity face the same health risks—some are metabolically healthy, while others with comorbidities such as diabetes or hypertension are considered metabolically unhealthy. This variation suggests that obesity classification needs to go beyond BMI and include more personalized assessments. Recent advances in body composition analysis, metabolic phenotyping, and technology—such as 3D scanning, smartphone-based tools, and machine learning—are improving our ability to define obesity subtypes and predict health outcomes. Integrating these tools into routine care could enhance prevention and management strategies for people living with obesity [5].

The growing prevalence of obesity worldwide has sparked renewed interest in understanding its underlying biological and physiological mechanisms. While the scale of the problem is well documented, the complexity of its causes and consequences continues to unfold. Advances in research have revealed that obesity is not a uniform condition, but rather a spectrum of phenotypes influenced by genetic, hormonal, environmental, and behavioral factors. Emerging methods—such as detailed body composition analysis, metabolic profiling, and genetic screening—are helping to better classify individuals with obesity and predict their risk for associated complications. These insights are also shaping new, more targeted treatment approaches, moving beyond traditional models focused solely on caloric imbalance. This paper explores the key mechanisms driving obesity, its systemic consequences, and the evolving strategies for its assessment and management.

## 2. Health Consequences of Obesity

### 2.1. The Impact of Obesity on Cardiovascular Diseases

Obesity promotes cardiovascular disease (CVD) through both direct and indirect mechanisms. Excess adipose tissue contributes to endothelial dysfunction, small vessel remodeling, and cardiomyocyte toxicity, which in turn promote the development of atherosclerotic and vasospastic coronary artery disease, arrhythmias, cardiomyopathy, and congestive heart failure (HF) [6,7].

Adipose tissue functions as a complex secretory organ, playing a key role in regulating energy expenditure, appetite, insulin sensitivity, inflammation, and immune responses. In individuals with obesity, adipose tissue becomes dysfunctional and secretes increased levels of pro-inflammatory proteins, such as interleukin (IL)-6, tumor necrosis factor alpha (TNF-α), C-reactive protein, and IL-18. In contrast, adipose tissue in lean individuals predominantly produces anti-inflammatory cytokines, including transforming growth factor beta, IL-4, IL-10, and IL-13. This shift toward a pro-inflammatory profile and an abnormal immune response in obesity significantly contributes to the development of chronic conditions such as hypertension, atherosclerosis, and HF [8,9].

#### 2.1.1. Obesity and Hypertension

The relationship between obesity and hypertension is well established, with estimates indicating that obesity accounts for approximately 65–78% of cases of essential hypertension [10]. The mechanisms underlying obesity-related hypertension include insulin- and leptin-mediated activation of the sympathetic nervous system, which in turn stimulates the renin–angiotensin–aldosterone system (RAAS) and promotes renal sodium retention. Additionally, adipose tissue directly contributes to RAAS activation by increasing the production of angiotensinogen, angiotensin II, aldosterone, and pro-inflammatory cytokines.

Enhanced renal sodium reabsorption shifts the pressure natriuresis curve to the right, meaning that higher arterial pressure is required to excrete sodium and maintain fluid balance. This explains the sodium sensitivity observed in many obese individuals with hypertension and often necessitates the use of diuretic therapy. These interconnected pathophysiological mechanisms help explain why hypertension is one of the most common and clinically significant cardiovascular complications associated with obesity [11].

#### 2.1.2. Obesity and Atherosclerosis

Dyslipidemia is a major contributor to the development of atherosclerotic CVD in individuals with obesity [12]. Numerous clinical trials, as well as Mendelian randomization studies, have demonstrated a causal role of apolipoprotein B (apoB)-containing lipoproteins in the initiation of atherosclerosis [13]. This process often begins early in life, with the uptake of cholesterol esters by macrophage-derived foam cells and their subsequent deposition within the vessel wall, leading to the formation of fatty streaks—a phenomenon that is accelerated by obesity-related insulin resistance (IR) and chronic inflammation.

Obesity is associated with the presence of overt atherosclerotic lesions even after adjusting for traditional metabolic risk factors such as hypertension, dyslipidemia, and hyperglycemia. Visceral adiposity, in particular, promotes systemic and vascular inflammation, which plays a key role in all stages of atherosclerosis, from the development of fatty streaks to plaque instability, rupture, and thrombosis [6].

IR, commonly seen in obesity, contributes to an atherogenic lipid profile characterized by elevated triglycerides, reduced high-density lipoprotein (HDL) cholesterol, and an increased proportion of small, dense low-density lipoprotein (LDL) particles. This dyslipidemia is a central component of the metabolic syndrome. Additionally, endothelial dysfunction driven by decreased nitric oxide bioavailability in the context of oxidative stress and inflammation further accelerates atherogenesis [14,15].

Carotid intima-media thickness, a recognized early marker of atherosclerosis, has been consistently associated with chronic obesity, particularly with elevated body weight persisting from childhood through adulthood. This emphasizes the long-term vascular consequences of sustained excess adiposity [6].

#### 2.1.3. Obesity and Heart Failure

HF and obesity are complex, frequently interrelated conditions. Obesity is present in approximately one-third of patients with HF and is even more prevalent among those with HF with preserved ejection fraction (HFpEF) [16,17]. Notably, the risk of incident HF increases by 41% for every 5-unit increase in BMI [18].

From a hemodynamic perspective, obesity leads to increased central blood volume and stroke volume, resulting in elevated cardiac output proportional to the degree of obesity. These changes promote structural cardiac adaptations, including left ventricle (LV) dilatation and compensatory hypertrophy. Additionally, obesity is associated with lower levels of brain natriuretic peptide, and the degradation of natriuretic peptides by adipose tissue contributes to plasma volume expansion and reduced ventricular compliance—key features in the pathophysiology of HFpEF.

Studies suggest that obesity-related HFpEF presents distinct phenotypic characteristics, such as more extensive biventricular remodeling, right ventricular dysfunction, impaired pulmonary vasodilation, pericardial restraint, and reduced exercise tolerance. Furthermore, atherosclerotic CVD associated with obesity may lead to LV systolic dysfunction, resulting in HF with reduced ejection fraction. Comorbid conditions commonly linked to obesity, such as obstructive sleep apnea (OSA) and obesity hypoventilation syndrome, further increase the risk of pulmonary hypertension and right ventricular failure [19].

Beyond structural abnormalities, metabolic dysfunctions induced by obesity—including IR and chronic systemic inflammation—play a significant role in the development and progression of HF [20]. These mechanisms underscore that obesity is not merely a risk factor but a central pathophysiological driver of multiple HF phenotypes. Therefore, addressing obesity is essential in both the prevention and effective management of HF and related cardiovascular diseases.

### 2.2. Metabolic Outcomes of Obesity

#### 2.2.1. Obesity and Insulin Resistance

Insulin is a peptide hormone secreted by pancreatic β-cells in response to circulating levels of glucose and fatty acids. Its primary metabolic role is the maintenance of glucose and lipid homeostasis. Following food intake, elevated blood glucose levels stimulate insulin secretion. Insulin exerts its effects by binding to receptors on target cells, particularly within skeletal muscle, adipose tissue, and the liver. This interaction facilitates glucose uptake into cells, where it is utilized for energy metabolism, fatty acid synthesis, and protein synthesis [21].

IR, also referred to as impaired insulin sensitivity, is characterized by a diminished biological response of target tissues—primarily the liver, muscle, and adipose tissue—to insulin stimulation [22]. In insulin-resistant states, cells fail to respond adequately to insulin, impairing glucose uptake and resulting in elevated circulating glucose levels. To compensate, pancreatic β-cells increase insulin secretion, marking the onset of hyperinsulinemia. As IR persists over time, β-cells are chronically stimulated to produce insulin in excess. This compensatory mechanism, sustained over months or years, leads to β-cell dysfunction and eventual exhaustion, contributing to chronic hyperglycemia [21].

Obesity is a major contributor to the development of IR, with over 80% of obese individuals exhibiting some degree of IR during their lifetime [23]. The pathophysiological link between obesity and IR involves adipocyte dysfunction and activation of the innate immune response. Adipocyte cell death in obesity releases damage-associated molecular patterns, including intracellular molecules and extracellular matrix components, which act as endogenous danger signals. These molecules activate Toll-like receptors, initiating an inflammatory cascade characterized by increased expression of pro-inflammatory genes aimed at tissue repair [24].

Notably, in humans, alterations in glucose metabolism—manifested by elevated blood glucose levels, reduced insulin sensitivity, and compensatory hyperinsulinemia—can be observed up to 13 years prior to the clinical diagnosis of T2DM [21].

#### 2.2.2. Obesity and Diabetes Mellitus 2

The global surge in obesity is undeniably contributing to the increasing prevalence of T2DM, a chronic and progressive metabolic disorder characterized by the body’s inability to produce sufficient insulin or to effectively utilize the insulin it produces. This dysfunction leads to elevated blood glucose levels (hyperglycemia), the hallmark of the disease. T2DM is now recognized as one of the most rapidly escalating global health emergencies of the 21st century [25].

Irrespective of the subtype, the sharp rise in obesity rates has been identified as a key factor in the parallel increase in diabetes mellitus (DM), which currently affects approximately 10.5% of the global population. Alarmingly, the incidence of DM among younger populations is also rising significantly [25,26]. T2DM, which accounts for over 90% of all diabetes cases worldwide, is strongly associated with overweight and obesity, aging, ethnicity, and a family history of diabetes. The disease is primarily driven by relative insulin deficiency caused by pancreatic β-cell dysfunction and peripheral IR [25].

While the immediate consequence of obesity may appear to be excessive weight gain, its long-term impact is more insidious. Progressive weight gain serves as a precursor to a variety of metabolic disorders, among which T2DM is particularly prominent. In T2DM, chronic hyperglycemia arises from reduced insulin sensitivity, primarily due to the loss of functional β-cell mass. Obesity significantly contributes to both the onset and progression of T2DM through mechanisms such as enhanced genetic and epigenetic susceptibility, microenvironmental changes that impair insulin signaling, β-cell dysfunction, and alterations in the microbiome–gut–brain axis.

The pathogenesis of T2DM also includes a substantial inflammatory component, which drives the progressive decline in β-cell insulin secretion alongside persistent IR [27]. This inflammation impacts early β-cell development and function, positioning overweight and obesity as potent accelerators of the disease process [28]. A significant proportion of individuals with obesity experience an intermediate phase known as “prediabetes” before progressing to overt hyperglycemia [29]. Although prediabetes is not classified as a distinct clinical entity, it represents a critical warning stage that warrants early intervention and comprehensive screening for T2DM and cardiovascular risk factors. Prediabetes is strongly associated with central obesity, hyperlipidemia, and hypertension [29].

In addition to those already diagnosed with T2DM, a large segment of the global population remains at high risk of future disease development due to impaired glucose tolerance or impaired fasting glucose. Notably, the progression to T2DM in these individuals is, in many cases, preventable through timely intervention. T2DM is both preventable and manageable through patient education, behavioral and lifestyle modifications, appropriate pharmacological treatment, and continuous medical support. Emerging evidence also supports the possibility of disease remission in certain cases [25]. However, the unpredictable nature of disease onset and the variable duration of the prediabetic phase mean that approximately 30–50% of individuals remain undiagnosed until complications arise and therapeutic intervention becomes necessary [25].

Obesity not only contributes to IR but may also precipitate early β-cell failure in some individuals with T2DM. Unfortunately, current pharmacological therapies have not demonstrated the ability to halt the progressive decline in β-cell function over time [26]. Interestingly, in a subset of individuals, T2DM may precede obesity. In these cases, inherent IR results in increased hepatic glucose production and compensatory hyperinsulinemia, which in turn may contribute to subsequent weight gain and the onset of obesity [30].

#### 2.2.3. Obesity and Dyslipidemia

Obesity, particularly central (visceral) adiposity, is strongly associated with the development of atherogenic dyslipidemia. This dyslipidemic profile is typically characterized by elevated plasma triglyceride levels, reduced concentrations of high-density lipoprotein cholesterol (HDL-C), and the predominance of small, dense LDL particles [31]. It is estimated that approximately 60–70% of individuals with obesity exhibit abnormal lipid profiles, including increased serum triglycerides, very low-density lipoproteins, apolipoprotein B, and non-HDL cholesterol, accompanied by reduced HDL-C levels.

Although total LDL cholesterol levels may not always be markedly elevated in individuals with obesity, a qualitative shift in LDL subfractions is frequently observed. Specifically, there is an increased prevalence of small, dense LDL particles, which are considered particularly atherogenic due to several properties: they are more susceptible to oxidative modification, exhibit enhanced uptake by macrophages, more readily penetrate the arterial intima, and demonstrate reduced affinity for LDL receptors, leading to prolonged plasma residence time [32,33].

The pathogenesis of these lipid abnormalities is multifactorial and closely linked to the metabolic disturbances characteristic of obesity. Central among these are increased fluxes of free fatty acids and triglycerides from hypertrophied adipose tissue to the liver, systemic IR, and adipocyte dysfunction. The latter is marked by reduced secretion of adiponectin and elevated levels of pro-inflammatory cytokines such as TNF-α and IL-6 [7,34]. This pro-inflammatory state contributes both to hepatic overproduction of atherogenic lipoproteins and impaired clearance of circulating lipids, thereby promoting the dyslipidemic phenotype commonly observed in obesity-related metabolic disorders [35].

Epidemiological studies consistently demonstrate that individuals with general or abdominal obesity have a significantly higher risk—estimated to be two- to threefold—of developing dyslipidemia compared to non-obese counterparts. These findings underscore the pivotal role of excess adiposity in the disruption of lipid metabolism and the consequent elevation of cardiovascular risk [33].

### 2.3. Systemic Effects of Obesity

#### 2.3.1. Correlation Between Obesity and Cancer Development

From an epidemiological perspective, individuals with obesity and diabetes exhibit a significantly higher risk and mortality rate for various types of cancer [36]. A growing body of evidence links adult obesity to an increased incidence of numerous malignancies, including endometrial, postmenopausal breast, ovarian, esophageal adenocarcinoma, gastric cardia, colorectal, pancreatic, liver, gallbladder, kidney, thyroid, and prostate cancers, as well as non-Hodgkin’s lymphoma and multiple myeloma [37,38]. Among these, the most pronounced associations are observed for endometrial and esophageal adenocarcinomas, where the relative cancer risk in obese individuals exceeds four times that of individuals with normal BMI [38,39,40].

Obesity profoundly alters the metabolic and functional landscape of adipose tissue. These changes disturb the secretion profile of the tissue, increasing the production of hormones, adipokines, inflammatory cytokines, growth factors, enzymes, and free fatty acids [41]. These secreted molecules—collectively known as adipose-derived factors—play a central role in cancer initiation and progression by promoting cellular metabolic reprogramming [42]. The role of dysfunctional adipose tissue in supporting tumorigenesis is increasingly recognized as a critical element in cancer biology.

Moreover, dynamic interactions between adipocytes and cancer cells further contribute to the remodeling of adipose tissue. This bidirectional crosstalk induces both morphological and functional changes, impairing normal endocrine and paracrine signaling [43]. In an obese state, adipose tissue homeostasis is disrupted, facilitating the release of molecules that fuel the heightened metabolic and energetic demands of cancer cells [42,44].

During direct interaction with tumor cells, adipocytes undergo phenotypic transformation, including delipidation, driven by cancer-derived bioactive factors. This process markedly reduces the expression of key adipocyte markers, such as adiponectin, leptin (LEP), and fatty acid-binding protein 4 [42,44]. As a result, adipocytes acquire a fibroblast-like morphology and are termed cancer-associated adipocytes (CAAs) [45]. Sustained exposure to tumor-derived paracrine signals stimulates CAAs to secrete free fatty acids, inflammatory mediators, adipokines, and growth factors. These factors collectively contribute to the formation of a tumor-supportive microenvironment that plays an essential role in cancer development and progression [42].

#### 2.3.2. Obesity Impact on Obstructive Sleep Apnea

Sleep deprivation has become increasingly prevalent in today’s around-the-clock society, with growing evidence indicating its significant impact on overall health. Among the most common and serious sleep disorders is OSA, which is estimated to affect nearly one billion individuals worldwide [46]. OSA is defined by recurrent collapse of the pharyngeal airway during sleep, resulting in impaired gas exchange—specifically hypoxemia and hypercapnia—and intermittent surges in catecholamines and other counter-regulatory hormones [47].

Obesity is a well-recognized and modifiable risk factor for OSA, playing a key role in both its development and severity. The prevalence of OSA among individuals with obesity is nearly twice as high as in those of normal weight [48]. Epidemiological data show that up to 40% of obese individuals, and approximately 70% of those diagnosed with OSA, are classified as obese [48,49].

This association is largely driven by both mechanical and metabolic consequences of excess adipose tissue. Fat accumulation in the cervical and abdominal regions increases upper airway resistance through direct anatomical compression and by reducing lung volume. These changes contribute to airway collapsibility during sleep by diminishing caudal traction on the upper airway structures [49].

In addition to mechanical effects, obesity induces hormonal and inflammatory alterations that further exacerbate OSA. Dysregulation of adipokines—particularly leptin, adiponectin, and ghrelin—affects respiratory control, energy balance, and systemic inflammation. Leptin resistance and decreased adiponectin levels, commonly observed in individuals with obesity, impair neuromuscular control of the upper airway, thereby contributing to the pathogenesis of OSA [48,49].

The clinical relevance of this relationship is underscored by findings from the SLEEP-AHEAD study, which revealed that 86.6% of obese patients with T2DM had OSA. Furthermore, clinically significant OSA, defined by an Apnea Hypopnea Index (AHI) score greater than 15 events per hour, was diagnosed in over half of this cohort (53.1%) [38,50].

Encouragingly, even modest weight loss has been shown to produce meaningful improvements in OSA severity. Reductions in body weight are associated with significant decreases in the AHI score, emphasizing the central role of weight management in the prevention and treatment of OSA [48].

#### 2.3.3. Impact of Obesity on Liver Disease Development and Progression

The global rise in obesity has been closely linked to an increasing prevalence of Metabolic Dysfunction-Associated Steatotic Liver Disease (MASLD), now considered the most common liver pathology associated with excess body weight [51,52]. MASLD represents a systemic metabolic condition frequently accompanied by IR, as well as both hepatic and systemic inflammation. Although simple hepatic steatosis remains its most prevalent form, approximately 10–20% of affected individuals develop non-alcoholic steatohepatitis, characterized by hepatic inflammation and progressive fibrosis. In a subset of patients, this condition can further evolve into advanced liver disease, including cirrhosis and hepatocellular carcinoma (HCC) [53].

In response to the multifactorial pathogenesis of fatty liver disease, the term metabolic-associated fatty liver disease has been proposed to more accurately encompass the metabolic dysfunctions underlying the condition [51,52]. Obesity is also recognized as an independent risk factor for HCC, particularly among individuals of European descent, with meta-analyses indicating that it approximately doubles the risk of developing this malignancy [54]. However, in the context of chronic hepatitis C virus infection, the relationship between obesity or BMI and HCC risk appears less consistent, with several studies reporting no significant association [55,56].

#### 2.3.4. The Impact of Obesity on Osteoporosis and Bone Mineral Density

For many years, obesity has been regarded as a protective factor against osteoporosis, largely due to its positive association with bone mineral density (BMD), particularly at weight-bearing sites such as the lumbar spine and femoral neck. This assumption has been incorporated into clinical risk assessment models, including the Fracture Risk Assessment Tool—FRAX, where higher BMI is associated with a reduced estimated risk of fracture [57].

However, emerging epidemiological and clinical data increasingly challenge this paradigm, suggesting that the relationship between obesity and bone health is more nuanced. While obesity may confer protection against certain fracture types, it may simultaneously increase the risk of others, depending on skeletal site and individual characteristics [58].

Biochemical studies indicate that obese individuals tend to exhibit lower levels of bone turnover markers—particularly those related to bone resorption—suggesting a reduction in bone remodeling activity [58]. One proposed mechanism involves the accumulation of adipose tissue within the bone marrow, a phenomenon that increases with age and systemic adiposity. This shift is thought to result from mesenchymal stem cells favoring adipogenic differentiation at the expense of osteogenesis [58,59,60].

Moreover, the distribution of adipose tissue appears to be a key determinant of its impact on bone. VAT has been identified as an independent negative predictor of both bone turnover and microarchitectural quality, even when controlling for total BMI. In contrast, subcutaneous fat may exert a neutral or even protective effect on skeletal integrity [61,62].

Adipose tissue-derived hormones also play a significant role in bone metabolism. In obese individuals, elevated estrogen levels—primarily resulting from increased aromatase activity in adipose tissue—are thought to reduce bone resorption and promote bone formation, thereby exerting a protective effect on BMD [63]. However, the role of leptin remains controversial. While in vitro studies suggest a stimulatory effect on osteoblast activity and inhibition of osteoclast genesis, in vivo findings in both animal models and humans have produced inconsistent results [64].

Adiponectin, another key adipokine, has been shown to exert a beneficial influence on bone through both in vitro and in vivo models. Notably, its levels are typically reduced in individuals with obesity, potentially diminishing its positive skeletal effects [60].

Although obesity is frequently associated with higher BMD, particularly in individuals with greater lean mass [65,66], this does not necessarily translate into improved bone strength or reduced fracture risk. The net effect of obesity on bone health is modulated by several interacting factors, including fat distribution, systemic inflammation, and hormonal milieu. As such, obesity should not be regarded as uniformly protective against osteoporosis.

## 3. Molecular Mechanism Underlying Obesity

### 3.1. Genetic Factors Associated with Obesity

#### 3.1.1. Polygenic Obesity and BMI

Despite decades of research, the underlying causes of obesity remain a topic of intense debate, often framed as the interplay between genetic predisposition and lifestyle factors. Genetic and epigenetic studies have provided a better understanding of the biological mechanisms underlying this phenomenon. One of the most popular indicators used to describe overweight is BMI [67].

A meta-analysis from 2022 has shown that BMI depends on additive genetic and environmental factors [67].

#### 3.1.2. Epigenetics and Environmental Influences on Gene Expression

Epigenetics involves changes in gene expression unrelated to DNA sequence modifications [68], but rather to processes such as DNA methylation, histone modifications, and microRNA activity [68,69].

Environmental factors, including maternal diet and exposure to toxins, have been shown to lead to permanent epigenetic changes and an increased risk of obesity in offspring [68,70].

Furthermore, diet or smoking can alter DNA methylation and histone modifications, impacting genes involved in appetite regulation, lipid metabolism, and insulin sensitivity [67].

#### 3.1.3. Polygenic Risk and Genome-Wide Association Studies

Obesity is usually polygenic in nature, which means that it depends on cumulating numerous genetic variants with small effects [71]. Modern GWAS studies have detected many single-nucleotide polymorphisms associated with obesity, which can be included in the group named polygenic risk score (PRS) [69,72]. Research shows that children with the highest PRS have a significantly increased risk of obesity, especially in unfavorable environments (e.g., lack of physical activity, diet) [69].

#### 3.1.4. Monogenic Forms of Obesity

Although rare monogenic obesity syndromes (e.g., those caused by mutations in the LEP gene, leptin receptor gene (LEPR), melacortin 4 receptor (MC4R), and fat mass and obesity-associated gene (FTO)) provide valuable information about the pathways regulating body weight [73,74]. Characteristic features include early onset, lack of satiety, and often autosomal recessive inheritance (as a Bardet–Biedel Syndrome) [73,74,75].

In some cases, causal treatment such as leptin therapy [73] or MC4R agonists is possible; this will be discussed in more detail in the following chapter.

The FTO gene is one of the first genes found to be associated with obesity [11,12]. Its relationship with BMI was primarily identified and published by GWAS in 2007 [76]. The rs9939609 polymorphism in intron 1 of the FTO gene has a strong association with BMI, body weight, and obesity risk in multiple populations. In the 2007 study by Frayling et al., adults homozygous for the risk allele (≈16% of participants) weighed on average about 3 kg more and had 1.67-fold increased odds of obesity compared to those without the allele [71].

The effect of FTO may be partially modulated by environmental factors such as physical activity and diet [73].

The MC4R is a brain-expressed G-protein-coupled receptor that regulates appetite in the hypothalamus [75]. Mutations in this gene are one of the most common causes of monogenic obesity, and together with the genes encoding leptin and LEPR, MC4R explains 30% of extreme obesity in children [73]. Those with an MC4R mutation often exhibit hyperphagia and weight gain in childhood [73].

Leptin is a satiety hormone produced mainly by adipose tissue at levels proportional to fat mass and encoded by the LEP gene [75]. Mutations in LEP or LEPR lead to severe, early-onset obesity [73,77].

Recombinant leptin treatment has been shown to be effective in patients with leptin deficiency [77].

### 3.2. Environmental Factors at the Molecular Level

#### 3.2.1. Environmental Contributions to Obesity Etiology

The etiology of obesity has traditionally been attributed to an imbalance between energy intake and expenditure, namely excessive caloric consumption and insufficient physical activity. However, this paradigm alone does not adequately account for the rapid global rise in obesity prevalence. Emerging evidence highlights the role of additional environmental factors, including alterations in gut microbiome composition [78], psychological stress [79], exposure to obesogenic chemicals [80], and dietary patterns [81], which collectively contribute to the complex pathophysiology of obesity.

#### 3.2.2. The Role of Obesogens

Obesogens are endocrine-disrupting chemicals with obesogenic potential [82]. These chemicals promote adipogenesis and fat storage, disrupt appetite regulation, or alter energy metabolism, predisposing individuals to obesity [83]. Common sources include industrial chemicals (e.g., Bisphenol A (BPA), phthalates, perfluorooctanoic acid), agricultural and marine chemicals (e.g., tributyltin), food additives, certain preservatives, artificial sweeteners, and packaging-related compounds [84].

Obesogens act through multiple mechanisms, such as binding to nuclear receptors (e.g., peroxisome proliferator-activated receptor gamma [PPARγ]), and are a key regulator of fat cell differentiation, thereby stimulating adipocyte formation [82,83]. Others disrupt hormonal signals, leading to disturbed energy balance, or promote epigenetic modifications, such as DNA methylation [83]. Obesogens may also alter gut microbiome composition [83].

The timing of exposure is particularly important. Sensitive windows such as prenatal life, childhood, and adolescence appear to heighten vulnerability [85]. Researchers have shown an association between prenatal exposure to BPA and increased adiposity in children [84,86].

#### 3.2.3. Dietary Patterns and Molecular Pathways

Another environmental factor related to obesity is a diet high in processed food, which is a source of obesogens (e.g., BPA) [85]. Furthermore, a high-fat diet activates inflammatory pathways (primarily nuclear factor kappa B), induces oxidative stress, and promotes IR [81,87]. Long-term consumption of simple sugars may lead to epigenetic modification of genes responsible for lipogenesis (e.g., SREBP-1c, FASN) and adipogenesis [85,88].

#### 3.2.4. Consequences of Gut Microbiota Dysbiosis

Gut microbiota dysbiosis refers to reduced microbial diversity and a decrease in bacteria-producing short-chain fatty acids (SCFAs) such as acetate, propionate, and butyrate; it leads to a weakened intestinal barrier, increased lipopolysaccharide penetration, and the activation of inflammation and IR [78,89].

SCFAs also perform signaling functions, including through GPR41/GPR43 receptors, influencing energy homeostasis and hormone secretion (GLP-1, PYY) [89]. Studies indicate that changes in the Firmicutes/Bacteroidetes ratio reduce the production of butyrate and other SCFAs, resulting in metabolic endotoxemia [78,87,88,89].

### 3.3. Insulin Resistance as a Pathophysiological Component

#### 3.3.1. Insulin Signaling Pathways

Insulin regulates systemic energy balance through distinct signaling cascades that control glucose, lipid, and protein metabolism. Upon insulin binding to its receptor, insulin receptor substrates (IRS) are phosphorylated and serve as key adaptor molecules that initiate downstream signaling [90,91]. Its primary actions are mediated by the PI3K-Akt pathway, which promotes glucose uptake, glycogen and lipid synthesis, and suppression of hepatic gluconeogenesis, and the Ras-MAPK pathway, which regulates growth and gene expression [90,91,92,93,94]. Crosstalk with nutrient and energy sensors, such as AMP-activated protein kinase (AMPK) and mTOR, further integrates anabolic and catabolic processes [80,95,96]. In insulin-sensitive WAT, the liver, and skeletal muscle, these pathways maintain metabolic flexibility and fuel homeostasis. However, selective impairment of IRS-PI3K-Akt signaling leads to reduced glucose disposal in muscle, enhanced lipolysis in WAT, and excessive gluconeogenesis in the liver, driving systemic IR [90,91,97,98]. The tissue-specific impairments and metabolic consequences are summarized in Table 1.

Understanding these tissue-specific mechanisms provides a foundation for targeted therapeutic strategies.

#### 3.3.2. Insulins’ Role in White Adipose Tissue, Skeletal Muscle, and Liver

In white adipose tissue (WAT), insulin’s principal role is to suppress lipolysis, thereby limiting the release of gluconeogenic substrates, notably free fatty acids, which otherwise fuel hepatic glucose production [92,95]. This antilipolytic effect is mediated through the PI3K-Akt pathway, which activates phosphodiesterase 3B (PDE3B), lowering intracellular cAMP and protein kinase A (PKA) activity [80,94]. Further modulation occurs via protein phosphatases such as PP1 and PP2A, which dephosphorylate lipolytic regulators, reinforcing insulin’s suppression of lipolysis [80,99]. Beyond curbing lipolysis, insulin promotes lipogenesis—predominantly in the liver, but also in WAT—by activating SREBP-1c, and indirectly, ChREBP, transcriptional regulators of genes that encode lipogenic enzymes, driving de novo lipogenesis [94,97]. This lipogenic program is complemented by PPARγ, the master regulator of adipocyte differentiation and lipid metabolism, which also enhances glucose homeostasis by increasing the expression of GLUT4 [100,101].

In skeletal muscle, the main site of insulin-mediated glucose uptake, insulin signaling promotes the translocation of GLUT4 vesicles to the plasma membrane [90,94,100]. PI3K regulates this process through Akt2 phosphorylation and the activation of the small GTPase Rac1 [80,100]. The key mechanisms and metabolic consequences are summarized in Table 1.

Concurrently, insulin enhances glycogen synthesis in both muscle and the liver by inhibiting glycogen synthase kinase-3 (GSK3) and activating PP1, resulting in dephosphorylation and activation of glycogen synthase [90,94,99]. It further suppresses glycogenolysis by downregulating phosphorylase kinase activity, thereby favoring glucose storage in muscle [90,91]. In insulin resistance, excess lipid accumulation within muscle cells generates diacylglycerols and ceramides, which activate novel PKC isoforms such as PKCθ [90,91]. These PKCs phosphorylate IRS-1 on serine residues, inhibiting its tyrosine phosphorylation and thereby impairing PI3K-Akt signaling, which reduces GLUT4 translocation and glucose uptake [90,91]. These tissue-specific impairments are summarized in Table 1.

In the liver, insulin inhibits glucose output by activating the insulin receptor tyrosine kinase, which phosphorylates IRS1/2, recruits PI3K, and activates Akt2 [80]. This signaling cascade suppresses hepatic gluconeogenesis primarily through phosphorylation of forkhead box O1 (FOXO1), promoting its exclusion from the nucleus and downregulating transcription of key gluconeogenic genes, including glucose-6-phosphatase and phosphoenolpyruvate carboxykinase [80,90].

A paradox of hepatic insulin resistance is that, while the insulin-resistant liver fails to suppress gluconeogenesis—contributing to hyperglycemia—it often remains sensitive to insulin’s lipogenic effects, promoting de novo lipogenesis and hepatic steatosis. This selective resistance arises from divergent intracellular signaling: impairment of the PI3K-Akt axis limits suppression of glucose production, whereas Akt-mTORC1-SREBP-1c signaling, which drives lipid synthesis, remains relatively intact. Clinically, this phenomenon underlies the coexistence of hyperglycemia, hyperinsulinemia, and MASLD in patients with metabolic syndrome and T2DM, as summarized in Table 1 [92,97,98].

**Table 1:** Summary of mechanisms and metabolic consequences of IR in WAT, skeletal muscle, and the liver. Abbrevations: DAG, diacylglycerol; PKC, Protein kinase C; IRS1, Insulin receptor substrate 1; PI3K, Phosphoinositide 3-kinase; Akt, Protein kinase B; PP1, Protein phosphatase 1; PP2A, Protein phosphatase 2A; FOXO1, forkhead box O1; mTORC1, mammalian target of rapamycin complex 1; SREBP-1c, Sterol regulatory element-binding protein 1; GLUT4, Glucose transporter type 4; FFA, Free fatty acids; MASDL, Metabolic Dysfunction-Associated Steatotic Liver Disease; ↑ increase; ↓ decrease.

Tissue	Mechanism of Signaling Impairment	Metabolic Consequences
Skeletal muscle	Lipid metabolites (DAG, ceramides) activate novel PKCs (e.g., PKCθ)→IRS1 serine phosphorylation → impaired PI3K–Akt signaling [90,91].	↓ GLUT4 translocation; ↓ glucose uptake
White adipose tissue (WAT)	(1) Impaired PI3K–Akt signaling [100,101]. (2) Altered phosphatase activity (PP1/PP2A)→ reduced suppression of lipolytic enzymes [80,99].	↑ Lipolysis; ↓ glucose uptake; ↑ FFA release → systemic insulin resistance
Liver	Selective insulin resistance: impaired PI3K–Akt signaling prevents FOXO1 suppression→ persistent gluconeogenesis [92]. Intact Akt–mTORC1–SREBP-1c signaling → excessive lipogenesis [97]. PKCε activation further inhibits insulin signaling [98].	↑ Hepatic glucose production; ↑ de novo lipogenesis (MASLD)

## 4. Stress-Related Mechanisms and Therapeutic Approaches in Obesity

### 4.1. Neuroendocrine Pathways Linking Stress and Obesity

#### 4.1.1. Hypothalamic–Pituitary–Adrenal Axis Activation in Stress-Related Obesity

Stress-related mechanisms in obesity have emerged as a growing field of research, with mounting evidence that chronic psychosocial stress not only influences eating behaviors but also alters neuroendocrine processes responsible for body weight regulation [79,101,102]. A central component of these mechanisms is the hypothalamic–pituitary–adrenal axis, whose prolonged activation leads to elevated cortisol concentrations [79,101].

#### 4.1.2. Metabolic and Appetite Consequences of Neuroendocrine Dysregulation

Persistently high cortisol promotes visceral adiposity, increases preference for calorie-dense foods, and disrupts satiety and hunger signaling [79,101]. Another important feature of stress-related obesity is the rise in circulating ghrelin following stress, which further drives comfort-food consumption [79,103], along with reducing leptin levels after stress—particularly pronounced in women—potentially impairing appetite suppression [102].

### 4.2. Psychotherapeutic Interventions for Stress-Related Obesity

#### 4.2.1. Stress-Related Barriers to Effective Weight Management

Standard weight-loss strategies that focus on caloric restriction and physical activity may fail in individuals experiencing chronic stress if the neuroendocrine drivers of appetite are not addressed [103]. An increasing number of researchers advocate for personalized interventions that integrate hormonal biomarker assessment with targeted behavioral therapies. Among the most frequently studied approaches are Cognitive Behavioral Therapy (CBT), Acceptance and Commitment Therapy (ACT), and mindfulness-based interventions such as Mindfulness-Based Stress Reduction (MBSR) [104,105,106,107,108,109].

#### 4.2.2. Efficacy of Behavioral Interventions (CBT, ACT, MBSR)

Meta-analyses confirm that CBT achieves modest but clinically relevant reductions in body weight, while improving cognitive restraint and reducing emotional eating [104,106]. CBT often outperforms standard behavioral therapy, particularly in maintaining weight loss over time [106]. ACT, which focuses on enhancing psychological flexibility and acceptance of unpleasant experiences, has been shown to improve psychological well-being in the majority of trials and, in some cases, to reduce body weight and alleviate eating-related difficulties [105]. Evidence also suggests that ACT can mitigate obesity-related stigma, thereby supporting long-term health behavior change [107]. Mindfulness-based programs, including MBSR, are effective in lowering perceived stress and biological markers such as cortisol and ghrelin [108,109], as well as reducing BMI and fat mass [108,110]. Clinical trials in pediatric populations have demonstrated that an eight-week mindfulness intervention combined with nutritional education leads to sustained reductions in body weight, body fat, and hunger and stress hormones [108]. In adults, mindfulness programs targeting stress-related eating have been associated with decreases in visceral adiposity and cortisol levels [109].

#### 4.2.3. Conclusions and Future Direction

The available evidence suggests that optimal treatment of stress-related obesity lies in integrating neuroendocrine assessment with individualized behavioral intervention selection. Individuals whose primary challenge is emotional eating may benefit most from CBT [104]; those with high levels of experiential avoidance may respond better to ACT [105,107]; and individuals with high stress reactivity may gain more from mindfulness-based programs [108,109,110]. Despite promising findings, there is a lack of randomized controlled trials directly testing biomarker-informed personalization strategies [111]. Future studies should stratify participants by hormonal profile to determine, with scientific rigor, which interventions are most effective for specific subgroups [104,107].

In conclusion, stress-related obesity requires a multifaceted approach that combines understanding of neuroendocrine mechanisms with evidence-based psychotherapeutic tools [79,101,103]. Integrating hormone profiling with targeted behavioral therapy may enhance treatment efficacy, and further research is needed to develop comprehensive, personalized therapeutic protocols supported by robust clinical evidence [104,107].

### 4.3. Therapeutic Use of Recombinant Hormones and Gene Therapy

Pharmacological agents approved for weight management have demonstrated both significant average and clinically meaningful reductions in body weight. Individuals with a BMI of ≥30 kg/m^2^, or ≥27 kg/m^2^ when accompanied by comorbidities such as T2DM, hypertension, dyslipidemia, OSA, or CVD, are considered suitable candidates for these therapies. Clinical trials have confirmed the efficacy and safety of these medications across diverse populations, including patients with various comorbid conditions and from a range of demographic, ethnic, and racial backgrounds [112]. Glucagon-like peptide-1 (GLP-1) is a peptide hormone composed of 30 or 31 amino acids, classified as a gastrointestinal hormone due to its secretion by the intestinal tract. It plays a key role in enhancing insulin secretion and suppressing glucagon levels under physiological conditions. Initially identified in 1983 as a cleavage product of proglucagon, GLP-1 is primarily secreted by enteroendocrine L cells in the gut, but also by pancreatic α-cells and neurons within the nucleus of the solitary tract. The actions of GLP-1 are mediated via its specific receptor, GLP-1R, a member of the G protein-coupled receptor family, which is expressed in pancreatic β-cells as well as in tissues such as the stomach, small intestine, mucosa, heart, and others. GLP-1 receptor agonists represent a class of pharmacological agents that act along the entero-insular axis. As a result, GLP-1R agonists are considered promising candidates for obesity treatment [113]. GLP-1 also plays a role in delaying postprandial gastric emptying and reducing gastric acid secretion. By inhibiting vagal nerve activity, GLP-1 suppresses gastric and duodenal motility and increases pyloric sphincter tone. These effects collectively lead to appetite suppression and body weight reduction, contributing to the so-called “ileal brake” phenomenon—where nutrient presence in the distal intestine slows gastrointestinal transit [114,115]. In experimental studies, Beiroa et al. demonstrated that administration of the GLP-1 analog liraglutide (LIR) in mice stimulated thermogenesis in brown adipose tissue and induced browning of white adipocytes, independently of food intake. LIR enhanced the utilization of triacylglycerol-derived fatty acids and glucose in brown adipocytes, leading to a reduction in lipid content. These effects were mediated through central GLP-1 receptor signaling pathways, involving key regulators such as AMPK and sirtuin-1, a NAD+-dependent deacetylase [116,117,118]. Semaglutide, a long-acting GLP-1 analog, is primarily used to manage T2DM but has also shown effectiveness in promoting weight loss in individuals with obesity. Administered via weekly subcutaneous injections, it has demonstrated significant weight reduction in both diabetic and non-diabetic patients. Clinical trials have confirmed that higher doses of semaglutide lead to more pronounced weight loss and a greater proportion of patients achieving clinically meaningful weight reduction. Like other GLP-1 receptor agonists, semaglutide’s main side effects are gastrointestinal, typically mild to moderate, and improve over time [113]. Its effectiveness has been confirmed in both people with and without T2DM, with added benefits such as improved glycemic control and reduced risk of obesity-related complications, including sleep apnea and progression to diabetes in individuals with prediabetes.

The most frequent side effects are mild to moderate gastrointestinal symptoms, especially in the first weeks of use. LIR also contributes to improved physical function and overall quality of life, especially in individuals achieving greater weight loss. Although dedicated cardiovascular outcome trials in people with obesity are still needed, current data suggest no increased cardiovascular risk [119].

One of the more recent concepts in modern medicine is the use of gene therapy as a potential approach to treating obesity. This method focuses on delivering and expressing specific therapeutic genes within targeted cells to restore and maintain energy balance. Several genes have been identified as key players in the regulation of metabolism and fat storage. These include circadian clock genes, β_3_-adrenergic receptor genes involved in thermogenesis and lipolysis, PPAR genes that guide adipocyte differentiation, the FTO gene associated with appetite and fat metabolism, LDL receptor genes, and glucocorticoid-related genes that contribute to visceral fat accumulation. Gene therapy strategies use different tools for gene delivery, including viral vectors (such as adenoviruses and adeno-associated viruses), nonviral carriers (such as peptides and lipids), and advanced genome-editing technologies, including zinc finger nucleases, CRISPR-Cas systems, and transcription activator-like effector nucleases (TALENs). Adenoviral vectors have been commonly used in animal studies but present certain challenges, including immune reactions, non-specific gene expression, and size limitations. In contrast, nonviral methods offer improved safety and better control over gene expression. Modern genome-editing technologies have significantly advanced the precision of genetic interventions. RNA interference, ZFNs, and TALENs allow for the silencing or correction of specific genes, although these approaches can be costly and technically complex. CRISPR-based systems, particularly CRISPR interference (CRISPRi) and CRISPR activation, offer precise control of gene activity using guide RNAs and a deactivated Cas9 enzyme to either suppress or promote gene transcription [120]. CRISPR interference (CRISPRi) works by utilizing a deactivated Cas9 (dCas9) enzyme, which is incapable of making double-stranded breaks in DNA but can still bind to target DNA sequences. This binding effectively blocks the transcription of specific genes by physically obstructing the transcription machinery or by recruiting repressive factors. The advantage of CRISPRi lies in its ability to subtly downregulate gene expression without permanently altering the genomic sequence, making it a reversible and precise tool for modulating gene activity. In the context of obesity, CRISPRi is employed to silence Fabp4, which regulates lipid binding and storage in adipocytes. By targeting Fabp4, researchers aim to modulate lipid metabolism and potentially reduce fat accumulation in white adipose tissue. One innovative approach in obesity research involves using a nonviral, adipose tissue-targeted gene delivery system to guide a CRISPRi complex toward white adipocytes, aiming to silence fatty acid-binding protein 4 (Fabp4)—a key regulator of lipid handling. In this method, an adipocyte-targeting peptide (ATS) fused to a 9-mer arginine sequence (ATS-9R) directs a catalytically inactive Cas9 (dCas9) plus Fabp4-specific sgRNA to white fat cells. The dCas9/sgRNA complex associates with ATS-9R via electrostatic binding, enabling selective uptake into adipose tissue and efficient nuclear delivery. On the other hand, CRISPR activation (CRISPRa) is a method used to enhance gene expression. It works through a similar system, but instead of the repressive proteins recruited by CRISPRi, CRISPRa uses a modified form of Cas9 that is tethered to transcriptional activators. This configuration can dramatically increase the transcription of a target gene by enhancing the recruitment of RNA polymerase or other activating factors to the gene’s promoter region. In obesity research, CRISPRa could be used to activate genes that promote fat breakdown, increase energy expenditure, or improve insulin sensitivity.

Both CRISPRi and CRISPRa offer powerful, complementary tools for gene regulation, enabling a fine-tuned approach to modifying gene expression. The ability to either inhibit or activate genes involved in lipid metabolism holds significant promise for advancing therapeutic strategies to combat obesity and related metabolic diseases. Furthermore, their application in adipose tissue—using nonviral gene delivery systems—ensures that these tools can be applied safely and effectively without the risks associated with viral vector-based methods.

Administered intraperitoneally twice weekly for six weeks in obese mice, this CRISPRi system achieved potent Fabp4 silencing, induced up to a 20% reduction in body weight, and alleviated IR and fatty liver disease. These findings suggest that precision-targeted CRISPRi to adipose tissue presents a promising therapeutic strategy for treating obesity and related metabolic disorders. For clinical translation, detailed pharmacokinetic, pharmacodynamic, safety, and dosage studies are required [121]. Recent advancements in FGF21-based gene therapy for obesity and insulin resistance have demonstrated encouraging results, showing the long-term potential of this approach. The gene therapy utilized AAV vectors to deliver the FGF21 gene to targeted tissues, including the liver and white adipose tissue. Remarkably, a single administration of these vectors led to sustained FGF21 expression, which resulted in significant and lasting reductions in body weight, improved insulin sensitivity, and decreased inflammation in adipose tissue. These benefits were observed in both young and older animals, with improvements lasting over a year. Additionally, the therapy effectively reversed liver damage and the development of MASLD, a common comorbidity of obesity and type 2 diabetes. Notably, the FGF21 gene therapy did not cause adverse effects such as bone loss or tumor formation, even when administered at higher doses. These results emphasize the efficacy and safety of FGF21 gene therapy, highlighting its potential as a long-term solution for treating metabolic disorders such as obesity and insulin resistance. Moreover, the therapy demonstrated favorable effects in healthy animals, promoting healthy aging without causing significant side effects, thus widening its potential therapeutic applications. This evidence paves the way for future clinical translation of FGF21 gene therapy to treat obesity, type 2 diabetes, and other metabolic diseases [122].

The potential of gene therapy in treating obesity has grown significantly in recent years. However, several limitations still exist, particularly related to the methods of gene delivery. Previous efforts have primarily focused on achieving stable and regulated expression of therapeutic genes using three main delivery methods: viral vectors, nonviral vectors, and physical methods. While viral vectors have remained the most commonly used due to their effectiveness, they raise concerns about safety in in vivo applications. On the other hand, synthetic vectors and physical methods present safer alternatives, although they tend to be less efficient in terms of gene delivery. Currently, nonviral approaches have not yet demonstrated full in vivo efficiency, except in cases of hydrodynamic gene transfer. Research continues to focus on improving gene delivery systems, aiming to create more efficient, safer, and targeted methods. One promising development in this area is the creation of artificial endonucleases with tailored specificity, which offer the possibility of avoiding random gene insertion. Unlike traditional gene therapy strategies that rely on random insertion of transgenes via viral vectors, these targeted approaches could potentially bypass the risks associated with such randomness. Additionally, further work is needed to develop custom-designed homing endonucleases that can efficiently target specific loci without causing toxicity. This innovation in genome-based editing presents a promising new avenue for overcoming current limitations in gene therapy. Another critical limitation to consider is maintaining sustained expression of the transgene. Gene silencing remains a major obstacle in gene therapy, but recent advances in promoter analysis and vector engineering offer hope for addressing this issue. Selecting the appropriate therapeutic gene for obesity treatment is also crucial, as obesity is a chronic, non-lethal condition, unlike genetic diseases related to single-gene deficiencies or cancer. Therefore, a careful evaluation of the long-term effects of gene transfer is essential to ensure the safety of therapeutic gene expression. Despite these challenges, it is clear that gene therapy could significantly influence the treatment of obesity and metabolic disorders in the future [123].

### 4.4. Role of Gut Hormones in Appetite and Weight Regulation

In humans, appetite is regulated by a complex interplay of neural signals, hormones, and external factors that control food intake. This regulation can be divided into central mechanisms—mainly involving the arcuate nucleus of the hypothalamus—and peripheral mechanisms, where hormones released from the gut and other organs play a key role. For the system to function properly, a fine balance between these signals is essential. Disruption of this balance can result in an energy imbalance, leading to weight gain and obesity. The human gut, being the largest endocrine organ, is highly metabolically active. It plays a vital role in maintaining energy balance by producing around 100 bioactive peptides and expressing over 30 genes responsible for gut hormone production. Together with peptides secreted by adipose tissue, gut hormones are crucial for regulating body weight by influencing both food intake and energy expenditure [124].

#### 4.4.1. Leptin and Ghrelin: Secretion Patterns and Physiological Roles

Leptin and ghrelin are two important hormones that have opposite effects on appetite and energy regulation. Ghrelin increases hunger and promotes food consumption by binding to specific receptors and activating appetite-stimulating neurons in the arcuate nucleus of the hypothalamus. In contrast, leptin works to decrease appetite and limit food intake [125]. In people with obesity, the blood level of leptin is elevated, while the level of ghrelin, which stimulates hunger, is unexpectedly reduced. However, obese individuals are resistant to leptin, meaning that despite high levels, its effects are diminished. Leptin is primarily produced by fat tissue, but small amounts are also generated in the stomach. This hormone influences many biological processes, such as puberty onset, immune responses, blood cell formation, wound healing, and bone growth. Once released into the bloodstream, leptin crosses the blood–brain barrier and binds to specific receptors in the hypothalamus. There, it influences various neurons and helps control the expression of neuropeptides that regulate food intake—decreasing the activity of orexigenic (appetite-stimulating) peptides such as neuropeptide Y and AgRP, while increasing anorexigenic (appetite-suppressing) peptides such as POMC and CRH. Leptin also helps counteract the action of ghrelin, the hunger-stimulating hormone, by interfering with the ghrelin-activated pathways in the hypothalamus. Through these mechanisms, leptin acts as a feedback signal that informs the brain about the body’s energy stores and contributes to the regulation of body weight. Additionally, gastric leptin—produced in the stomach—may play a role in short-term appetite control and meal size, especially in response to gut hormones and insulin released after eating [126]. Leptin resistance limits the effectiveness of leptin therapy for obesity, but targeting leptin receptors and downstream signaling in specific brain regions may help. One approach is improving leptin delivery across the blood–brain barrier (BBB). However, many strategies fail due to BBB alterations in obesity. Modified forms of leptin, such as Tat-leptin or PASylated leptin, and synthetic leptin-like molecules with better brain access and stability, show promise in animal models. Conventional leptin therapy has limited effects in humans, but combining leptin with other hormones such as amylin, GLP-1, CCK, or insulin has shown stronger weight-loss effects in animals. However, one promising combo—leptin with pramlintide—was halted due to antibody development. Targeting leptin signaling regulators such as suppressor of cytokine signaling 3 (SOCS3) and protein tyrosine phosphatase 1B (PTP1B) could improve leptin sensitivity. Inhibiting PTP1B, for instance, has reduced body weight and improved metabolism in animal studies. Drugs such as trodusquemine cross the BBB and show promising results. Intranasal leptin delivery is another potential method, shown to reduce appetite and liver fat in obese rats, but practical challenges remain, such as cost and inconsistent absorption. Interestingly, high leptin levels in obesity may actually worsen leptin resistance. Lowering leptin to physiological levels using anti-leptin antibodies in animals has reduced food intake and body weight, suggesting a novel therapeutic direction. Overall, restoring leptin sensitivity through improved delivery methods, combination therapies, or targeting leptin signaling pathways, offers promising strategies for treating obesity, although clinical challenges remain [127,128].

Ghrelin is mainly produced by enteroendocrine cells in the stomach’s fundus, as well as in the intestine, pancreas, and brain, including the arcuate nucleus (Arc) of the hypothalamus. Ghrelin becomes biologically active when acylated by the enzyme GOAT, forming acyl-ghrelin. Ghrelin levels rise before meals, drop after eating, and gradually increase again. These fluctuations are closely linked to hunger sensations. In animal studies, ghrelin injections into the Arc stimulate appetite and influence neurons involved in feeding behavior. Ghrelin may also act centrally by reducing serotonin-related appetite suppression. Although earlier studies suggested the vagus nerve was necessary for ghrelin’s effect on eating, later research indicates a direct action in the brain. Ghrelin may impact blood sugar control by speeding up gastric emptying, reducing insulin secretion, and promoting glucagon release. Fasting ghrelin levels are lower in obesity but increase with weight loss. Insulin may suppress ghrelin, partly explaining this pattern. Ghrelin secretion rises during fasting and is inhibited by meal-related reflexes. While ghrelin levels correlate with hunger and meal size, its direct role in causing hunger remains unclear. It may help prepare the body for nutrient intake and storage. Research in obese individuals and those undergoing Roux-en-Y gastric bypass (RYGB) has shown mixed results. Ghrelin-targeting drugs are under development and may clarify ghrelin’s role further. Due to ghrelin’s role in stimulating appetite and fat storage, antagonists or inverse agonists have been considered as potential treatments for obesity. However, research results are inconsistent—some studies report reduced food intake and weight loss, while others show the opposite effect. This highlights the complexity of ghrelin’s action and the need for further investigation before such therapies can be reliably applied [129].

#### 4.4.2. Peptide YY and Appetite Suppression

Peptide YY (PYY) is a hormone mainly produced by open-type enteroendocrine cells in the distal small intestine and colon. These cells often also secrete GLP-1 and sometimes other gut hormones. PYY exists in two main forms: PYY(1–36), the initially secreted form, and PYY(3–36), the active form created by enzymatic cleavage. PYY is also found in the pancreas and brain. PYY levels rise about 15–30 min after eating, peak after 1–1.5 h, and stay elevated for several hours. PYY(3–36) is thought to reduce appetite, although human studies show inconsistent results. While intravenous PYY(3–36) strongly reduces food intake in animals, this effect is less clear and possibly non-physiological in humans. Central administration of PYY(1–36) in animals can even increase eating. PYY may help regulate blood sugar by slowing gastric emptying, improving insulin sensitivity, and supporting pancreatic cell function. However, PYY(3–36) does not seem to directly affect insulin secretion in humans. Obesity’s impact on PYY secretion is unclear. Some studies report lower fasting and post-meal PYY levels in obesity, while others do not. Responses also vary by age and weight-loss status. PYY(3–36) is released in response to the digestion of all macronutrients, but this response may be reduced in people with T2DM. Overall, while PYY(3–36) may influence appetite, gastric emptying, and glucose control, its exact physiological role remains uncertain, partly due to research challenges such as side effects from infusion and limited tools for studying its receptor pathways [130]. Early studies showed that peripheral PYY(3-36) administration reduced food intake and body weight in both animals and humans, but its short half-life and side effects, such as nausea, limited its clinical use. Subcutaneous and intranasal delivery had limited success, with high doses poorly tolerated. As a result, efforts have shifted toward developing PYY(3-36) analogs with improved stability and reduced side effects. Some analogs, especially when combined with semaglutide, have shown promising results in animals. However, translating these findings to humans remains difficult due to species differences and past trial discontinuations [129].

#### 4.4.3. Glucagon-like Peptide-1 in Metabolic Control

GLP-1 is produced in the gut, brainstem, and to a lesser extent in the pancreas. It acts through a single receptor, GLP1R, to regulate energy balance. Research has identified several brain regions with GLP1R that are important for controlling food intake and responding to GLP-1 receptor agonists (GLP1RAs). These effects are largely mediated by the central nervous system via adrenergic and AMPK pathways, which stimulate brown adipose tissue (BAT) activity. When GLP1R is selectively reduced in the hypothalamus, animals show lower BAT activity, reduced energy expenditure, and increased weight gain. Although gut-derived GLP-1 is present in low concentrations in the blood, its absence does not lead to increased food intake or weight gain in mice, even with high-fat diets. This suggests that gut-derived GLP-1 is not essential for weight regulation. GLP-1 receptors are widely distributed in the brain, including in areas such as the hypothalamus, brainstem, and amygdala. Studies in rodents show that central administration of GLP-1 suppresses appetite, and this effect disappears in GLP1R-deficient mice or when blocked by an antagonist. In humans, GLP-1 or GLP1RA administration increases satiety and reduces hunger. Brain imaging studies show that GLP-1 reduces activity in brain regions associated with reward and food motivation, such as the amygdala, insula, and orbitofrontal cortex. Similar brain responses occur during food intake and GLP-1 infusion. In people with obesity, GLP1RA treatment, such as exenatide, alters brain connectivity in the hypothalamus, and these changes are linked to differences in appetite reduction between individuals. These effects are seen both in those with and without T2DM [114]. Between 2012 and 2014, several centrally acting drugs, including GLP1RAs such as LIR and the combination of naltrexone and bupropion, were approved for long-term weight management. In 2021, semaglutide 2.4 mg weekly was added, showing nearly double the weight loss seen with earlier treatments, marking a major advance in obesity therapy. The later approval of oral semaglutide further expanded options for patients, particularly those with T2DM seeking non-injectable treatments. GLP1RAs not only improve glycemic control and support weight loss in people with and without diabetes, but they may also offer long-term cardiovascular, renal, and metabolic protection. These benefits, along with sustained weight loss and low risk of rebound, support broader use of GLP1RAs in obesity treatment. However, more real-world studies are needed to confirm their long-term safety, effectiveness, and impact on quality of life. GLP-1 may also influence reproductive and inflammatory processes, suggesting a broader role in managing metabolic and hormonal imbalances. Despite promising data, the complexity of GLP-1’s effects highlights the need for further research, especially in real-life settings and comorbid conditions such as fatty liver disease [131].

#### 4.4.4. Cholecystokinin and Satiety Signaling

Cholecystokinin (CCK) is released by intestinal I-cells in response to dietary fats and proteins, through receptors such as GPR40 and the calcium-sensing receptor. It acts mainly on vagal afferent neurons, reducing food intake and slowing gastric emptying. CCK also stimulates pancreatic enzyme release and gallbladder contraction. CCK affects vagal neurons both by immediately altering their activity and, over time, by changing receptor and neurotransmitter expression—effects enhanced by leptin and blocked by ghrelin. In obesity, vagal neurons become less responsive to CCK, leading to reduced satiety signaling and altered energy balance. CCK also activates specific brain regions such as the hypothalamus and brainstem, especially after fat ingestion, and this brain response is blocked by CCK-1 receptor antagonists. Although CCK contributes to satiety and may support pancreatic function, its effects are blunted in obesity due to reduced vagal sensitivity and lower neuronal excitability [132]. Building on the success of GLP-1–based drugs, researchers have developed enzyme-resistant and long-acting CCK analogs—such as glycated CCK-8, (pGlu-Gln)-CCK-8, and PEGylated versions of CCK-8, CCK-9, and CCK-10—to improve stability and effectiveness. In obese and diabetic rodent models, these modified peptides reduced appetite, body weight gain, blood glucose, and lipid levels. Although PEGylation improved pharmacokinetics, it did not significantly enhance efficacy beyond the native modified peptide. Combining CCK with other hormones such as GLP-1, amylin, or leptin shows promise for synergistic effects in treating obesity and T2DM, highlighting the therapeutic potential of CCK-based strategies [133].

#### 4.4.5. Bariatric Surgery and Gut Hormone Modulation

Bariatric surgery is a highly effective treatment for obesity, leading to significant and lasting weight loss, along with beneficial metabolic changes. Procedures such as gastric banding, sleeve gastrectomy (SG), and RYGB alter gastrointestinal structure and function, influencing hormone responses and nutrient absorption. Gastric banding is adjustable, safe, and minimally invasive. SG removes about 80% of the stomach, preserving the pylorus, and has become widely used due to its strong efficacy and low complication rate. RYGB creates a small gastric pouch that bypasses part of the small intestine, offering the greatest weight loss but with a higher risk in modified versions with shorter common limbs [134]. Bariatric surgery significantly alters endocrine function due to structural changes in the gastrointestinal tract and new nutrient pathways. These changes may increase pancreatic sensitivity and impact various hormones, including GLP-1, ghrelin, CCK, PYY, TSH, and T3, with notable reductions in thyroid hormones. GLP-1 plays a key role in linking hormonal shifts to glucose regulation. Other effects include increased bone turnover, potential hypothalamic inflammation, and improved insulin sensitivity and beta-cell function. However, some of these mechanisms remain under debate [135]. Bariatric surgery, particularly Roux-en-Y gastric bypass, enhances incretin response—especially GLP-1 secretion—leading to improved oral glucose tolerance and insulin sensitivity. Studies show that GLP-1 levels rise significantly after RYGB, regardless of whether nutrients are delivered via the stomach or jejunum, likely due to increased exposure of distal GLP-1—producing cells to nutrients. Research has also highlighted the role of bile acids in post-surgery metabolic improvements. After RYGB, serum bile acid levels rise and correlate with improved insulin sensitivity. In mouse models, diverting bile to the ileum mimicked many RYGB benefits, such as reduced weight, improved glucose tolerance, and increased GLP-1 secretion—especially under low-fat diet conditions. These effects depended on the presence of GLP-1 receptors. Additionally, novel findings suggest that gut immune cells (β7+ intraepithelial lymphocytes) help regulate GLP-1 availability. Mice lacking these cells showed higher circulating GLP-1, were resistant to metabolic diseases, and had better glucose control, indicating that GLP-1 receptor signaling in the gut plays a broader role in metabolic regulation [136]. Recent studies have explored the role of ghrelin in the weight-loss effects of bariatric surgery, especially sleeve gastrectomy. A meta-analysis confirmed a significant ghrelin drop after SG, while results after Roux-en-Y gastric bypass remain inconsistent. Despite their different mechanisms—SG being restrictive and RYGB combining restriction with malabsorption—both procedures yield significant weight loss. Notably, SG patients show a marked and lasting decrease in ghrelin and appetite, suggesting this may compensate for the absence of malabsorption. A study by Karamanakos et al. confirmed sustained appetite suppression and ghrelin reduction in SG patients, unlike those who underwent RYGB [137]. Studies have shown that postprandial CCK levels rise after RYGB, despite the duodenum—normally the main site for CCK release—being bypassed. This may be due to alternative stimulants such as parasympathetic input or increased CCK cell activity in the distal intestine. While elevated CCK might aid satiety and glucose control, some data suggest higher CCK levels in poor RYGB responders. Comparatively, sleeve gastrectomy leads to a greater and more sustained increase in CCK than RYGB, while the impact of gastric banding on CCK remains unknown. Obesity is linked to lower postprandial PYY3-36 levels, while PYY3-36 infusion reduces food intake. After bariatric surgery, postprandial PYY levels rise across various procedures—including gastric banding, sleeve gastrectomy, and RYGB—with effects visible within weeks and lasting at least a year. Animal studies suggest PYY plays a key role in surgery-induced weight loss [138]. Each bariatric procedure results in a distinct pattern of gut hormone changes, with Roux-en-Y gastric bypass showing particularly strong synergistic effects that promote satiety and reduce food intake. Among these, GLP-1 and PYY3-36 have the most robust evidence supporting their role in suppressing appetite after surgery. Additionally, early postoperative increases in hormones such as glicentin and oxyntomodulin after both RYGB and sleeve gastrectomy are associated with more favorable weight-loss outcomes and could serve as early indicators of insufficient weight reduction, helping to identify patients who may need extra support. GLP-1 also contributes to improved insulin secretion shortly after surgery, even in individuals without T2DM, and may be involved in the development of postprandial hypoglycemia. Ongoing studies are testing GLP-1 receptor antagonists such as Ex-9 as potential treatments for this condition. Based on these insights, combinations of gut hormone receptor agonists are being developed to replicate the beneficial hormonal profile seen after bariatric surgery, offering promising new treatment strategies for obesity and T2DM [119].

The regulation of appetite and energy balance involves a complex interaction between central and peripheral signals, in which gut-derived hormones such as leptin, ghrelin, GLP-1, PYY, and CCK play critical roles. While hormonal therapies targeting these pathways have shown varying degrees of success, their efficacy is often limited by compensatory mechanisms and physiological resistance, as seen in leptin resistance or ghrelin’s inconsistent effects. Bariatric surgery emerges as the most effective long-term intervention for obesity, not only through anatomical changes but also by inducing favorable hormonal shifts—particularly increased levels of GLP-1 and PYY—that enhance satiety and improve glucose metabolism. These findings support the development of combination therapies and hormone analogs that mimic the post-surgical endocrine environment, offering a promising direction for future obesity treatment strategies.

## 5. Potential Preventive Strategies

### 5.1. Circadian Mislignment and Its Role in Obesity

The molecular circadian system consists of key proteins such as CLOCK, BMAL1, PER, and cryptochrome, which interact through transcription–translation feedback loops to generate self-sustained circadian rhythms within individual cells [139]. At the cellular level, core clock genes regulate the rhythmic expression of thousands of output genes that control various physiological processes according to the day–night cycle. This mechanism is driven by the CLOCK and BMAL1 proteins, forming a dimer that binds to E-box DNA elements, activating genes such as PER and CRY. As PER and CRY proteins accumulate, they translocate to the nucleus and inhibit CLOCK/BMAL1 activity, reducing their own expression. After PER and CRY degradation, the cycle restarts every 24 h. Additionally, CLOCK/BMAL1 activity regulates other transcription factors such as RORs and REVERBs, which form a secondary feedback loop fine-tuning BMAL1 expression, together controlling rhythmic activity of up to 25% of human genes [140].

In healthy individuals, maintaining stable blood glucose levels requires coordinated actions of several organs. The pancreas regulates blood sugar by releasing insulin and glucagon, the liver adjusts glucose production and storage, and muscles and fat tissues absorb glucose from the bloodstream under insulin’s influence. Additionally, incretin hormones such as gastric inhibitory polypeptide and GLP-1, secreted by the gut in response to glucose, help enhance insulin secretion. This complex system is tightly controlled by the body’s internal circadian clock, which not only dictates when we eat but also times insulin release, liver glucose metabolism, muscle glucose uptake, and incretin secretion. Research in rodents shows that eating at times misaligned with the body’s internal clock disrupts synchronization between central and peripheral clocks, as well as between different tissues and gene rhythms within organs. Light is the primary environmental cue that synchronizes the circadian clock, which in turn coordinates peripheral clocks throughout the body via hormonal and neural signals. Furthermore, light feeding and fasting cycles are key external factors that synchronize many peripheral clocks [141].

In vivo studies in mice have demonstrated that IR is linked to reduced activity of the Akt signaling pathway, which also plays a role in circadian regulation. In Bmal1 knockout mice, insulin-stimulated Akt phosphorylation in liver and muscle is significantly diminished, correlating with impaired glucose metabolism. Transgenic expression of Bmal2 restores Akt phosphorylation in the liver, particularly at the S473 residue, indicating reactivation of this pathway and improved insulin sensitivity. However, Akt phosphorylation in muscle remains largely unaffected, likely due to low Bmal2 expression in that tissue. Since liver and adipose tissue are more insulin-sensitive, the low insulin dose used in the experiments revealed these improvements. These findings suggest that restoring circadian rhythms via Bmal2 expression is linked to the recovery of normal metabolic function in B1ko mice [139].

Furthermore, a study on 14 adults simulating shift work over six days showed that working and eating during the biological night—when the internal circadian clock promotes sleep—disrupts the alignment of behavioral and metabolic rhythms regulated by clock genes such as CLOCK and BMAL1. This circadian misalignment leads to altered energy metabolism and increases the risk of obesity. On the first nightshift day, total daily energy expenditure slightly increased due to an afternoon nap and extended wakefulness, but decreased by about 3% on subsequent nightshift days. Energy expenditure dropped significantly during scheduled daytime sleep despite disturbed sleep quality. The thermic effect of food after a late dinner was also reduced, indicating less calorie burning post-meal. Initially, fat utilization increased during night shifts, but carbohydrate and protein utilization declined later. Interestingly, hunger ratings decreased, and appetite-suppressing hormones such as leptin and peptide YY were lower. These findings suggest that circadian disruption caused by shift work impairs the normal functioning of molecular clocks, which in turn dysregulates metabolic processes and contributes to weight gain [142]. In another study, it was shown that circadian misalignment in chronic night shift workers leads to increased levels of acylated ghrelin, a hormone that stimulates appetite, and heightened feelings of hunger in the morning. Although energy expenditure and metabolism remained largely unchanged, participants exhibited greater physical activity despite feeling sleepier. These results indicate that disruption of the circadian rhythm can alter hunger regulation, potentially contributing to the increased risk of obesity observed in shift workers [143]. It has been shown that light therapy can influence circadian misalignment and sleepiness in shift workers. Specifically, moderate-intensity light exposure for shorter durations with multiple daily sessions appears to be more effective in reducing sleepiness, while higher-intensity light is more successful in shifting the circadian phase. These effects vary depending on the type of shift work, with night shift workers benefiting more than rotating shift workers. Light therapy may help suppress melatonin secretion and adjust the sleep–wake cycle, improving alertness during work and facilitating better sleep timing. Despite some methodological limitations in the studies analyzed, these findings suggest that appropriately timed and dosed light therapy could be a valuable tool in managing circadian disruption and its metabolic consequences among night shift workers. These findings may have important implications for preventing obesity in night shift workers by improving circadian alignment and reducing sleepiness-related metabolic disruptions [144]; timed light therapy can significantly improve blood pressure regulation and glucose tolerance, even without changes in melatonin or cortisol levels. These improvements were associated with reduced catecholamine levels, suggesting that light therapy may be a promising intervention to counteract the metabolic disturbances caused by circadian disruption—offering real potential in the prevention and treatment of obesity among night shift workers [145].

### 5.2. Molecular Understanding of Parental Obesity Influence on Offspring

Parental obesity has been shown to influence the health and metabolism of offspring through epigenetic modifications. Studies indicate that both paternal and maternal obesity can lead to changes in DNA methylation patterns in newborns, which may affect gene expression related to growth and metabolic regulation. These epigenetic alterations potentially contribute to the child’s risk of developing obesity and metabolic disorders later in life. Understanding how parental health impacts offspring at the molecular level is essential for developing early interventions to reduce obesity risk across generations [146]. Environmental factors can induce changes in DNA methylation patterns in the germline, leading to heritable epigenetic modifications known as germline epimutations. These alterations have the potential to influence the transcriptome and epigenetics of totipotent cells in the early embryo, which subsequently affect all somatic cells derived from these stem cells. Such environmentally induced epigenetic changes can disrupt the normal cascade of gene expression and epigenetic programming during early development, increasing susceptibility to diseases such as obesity later in life. Epigenetic transgenerational inheritance occurs when these epigenetic modifications are passed through multiple generations without continued direct environmental exposure. This process can begin when environmental insults affect a gestating female (F0), impacting not only her fetus (F1) but also the germ cells within that fetus, which give rise to the F2 generation. If the altered epigenetic patterns persist into the F3 generation, this is considered true transgenerational inheritance. Similarly, preconception exposure to environmental factors can modify the germline epigenome of the F0 generation, affecting subsequent generations beyond those directly exposed. In such cases, the first generation not directly exposed to the environmental insult—F3 in gestational exposure or F2 in preconception exposure—represents the transgenerational offspring. These integrated genetic and epigenetic changes during critical developmental windows help establish disease susceptibility, including obesity risk, across generations [147]. Epigenetic modifications are stable and inheritable changes to DNA that do not alter the genetic code itself. These modifications include DNA methylation, histone post-translational modifications, and regulation by noncoding RNAs such as microRNAs, which influence gene expression at transcriptional and post-transcriptional stages. DNA methylation depends on the availability of methyl groups supplied by one-carbon metabolism, which can be disrupted by micronutrient deficiencies often linked to obesity. For example, obesity is associated with deficiencies in folate and vitamin B12 and increased homocysteine levels in humans. In mouse studies, a high-fat diet reduces methyl donor availability, leading to altered epigenetic programming. Similarly, vitamin B12 deficiency during human adipocyte differentiation changes microRNA expression related to fat cell function, affecting the release of adipocyte-derived circulating microRNAs that regulate adipogenesis [148,149,150]. Vitamin D also plays an important role in epigenetic regulation. Due to increased fat mass, individuals with obesity frequently experience vitamin D deficiency. Offspring of vitamin D-deficient mothers or vitamin D-deficient mice tend to have higher BMI. Furthermore, insufficient vitamin D during childhood is associated with greater adiposity and metabolic syndrome later in life [151,152]. During pregnancy, the mother’s body undergoes important metabolic changes to support the growing fetus and prepare for breastfeeding. In women with a healthy weight, these changes happen smoothly: early in pregnancy, insulin sensitivity and adiponectin levels increase to promote fat storage mainly in the lower body. Later, IR develops to provide more energy and nutrients to the fetus. However, in women who are already overweight or obese before pregnancy, these normal adaptations are disrupted. Their fat tissue, especially around the abdomen and upper body, is metabolically unhealthy, showing increased leptin and extracellular vesicles (EVs) and decreased adiponectin. This unhealthy fat distribution leads to greater IR and reduced ability for fat storage expansion. As a result, obese pregnant women are at higher risk for metabolic problems such as gestational diabetes and high blood pressure. The placenta in these pregnancies also shows dysfunction, releasing more leptin, inflammatory cytokines, and EVs, which increases nutrient supply to the fetus. This environment promotes excessive fetal growth and fat accumulation, increasing the child’s risk of developing obesity, hypertension, and diabetes later in life [153]. As obesity rates continue to rise, more pregnancies are affected, increasing the likelihood of metabolic disorders in children. Gaining insight into how parental obesity influences metabolic programming in offspring—leading to a higher risk of metabolic syndrome in adulthood—is essential for developing effective interventions to interrupt this cycle.

### 5.3. Effects of Maternal Smoking on Childhood Obesity

Prenatal exposure to maternal tobacco smoking remains a major contributor to birth complications and is also linked to developmental impairments in children. Among the potential mechanisms, epigenetic alterations—such as changes in DNA methylation, histone modification, and microRNA expression—may explain how smoking during pregnancy leads to negative outcomes [154]. Despite growing evidence, the exact mechanisms by which early-life exposure to tobacco smoke affects adipose tissue development and hormonal function remain unclear. Increased adiposity is commonly linked to elevated leptin levels. Leptin’s role in energy homeostasis may also involve the hypothalamic–pituitary–thyroid axis and thyroid hormone metabolism [155]. Disruptions in thyroid function can significantly alter energy expenditure and body weight, as leptin and thyroid hormones influence each other. Tobacco use is known to affect thyroid activity, although thiocyanate—a compound found in tobacco smoke rather than nicotine—is more often linked to thyroid dysfunction such as hypothyroidism and goiter [156,157].

Maternal smoking may impact newborn thyroid function differently depending on maternal iodine levels. In mothers with adequate iodine intake, smoking can suppress TSH and cause neonatal hyperthyroidism, whereas in iodine-deficient mothers, smoking is associated with goiter in infants. Smoking also reduces iodide content in breast milk, resulting in lower urinary iodide levels in infants, suggesting impaired iodide transfer due to nicotine [158,159]. Animal studies further support these findings. In a rodent model, nicotine-exposed mothers showed reduced T4 and mammary gland iodide uptake, along with increased TSH levels. Their offspring had low thyroid iodide uptake and altered thyroid hormone levels. Interestingly, after nicotine exposure ceased at weaning, pups regained normal thyroid function, likely due to restored T3 transfer via lactation. These results suggest that early-life nicotine exposure disrupts thyroid function in both mother and offspring, potentially through impaired leptin signaling. Persistent thyroid dysfunction in adulthood may reflect long-term leptin resistance, both centrally and peripherally [160].

Maternal smoking during pregnancy increases oxidative stress in newborns due to elevated levels of free radicals and immature antioxidant systems. As a result, offspring of smoking mothers show reduced levels of glutathione—a key antioxidant protecting against oxidative damage—and decreased activity of enzymes that neutralize oxidative stress. This deficiency in protective mechanisms makes neonatal tissues more vulnerable to damage, potentially disrupting the normal function of cells involved in metabolism and energy regulation. Such damage and chronic oxidative stress can program adverse metabolic changes, increasing the risk of obesity development later in the child’s life [161]. Additionally, nicotine impacts pancreatic development by inducing oxidative stress that causes beta-cell damage, impairing insulin production and glucose regulation. These changes promote metabolic disturbances such as IR and dysregulated energy homeostasis. Epigenetic modifications, including DNA methylation and histone changes triggered by oxidative stress and altered glucocorticoid signaling, further program gene expression patterns that predispose offspring to obesity and metabolic syndrome later in life. Thus, prenatal nicotine exposure initiates molecular and cellular alterations through oxidative stress and epigenetic reprogramming that increase the risk of obesity in children [162].

## 6. Emerging Technologies for Obesity Management—Artificial Intelligence-Powered Systems

### 6.1. Role of Artificial Intelligence in Personalized Obesity Management

Artificial intelligence (AI) has seen rapid growth in recent years, becoming a driving force of innovation across multiple fields, with healthcare standing out as a key area for its transformative impact. By utilizing large-scale, long-term patient datasets, AI has the potential to reshape both clinical care and administrative processes. AI approaches—such as machine learning (ML), deep learning, and natural language processing—offer a wide range of capabilities. When effectively integrated into health systems, AI can enhance diagnostic accuracy, support evidence-based decision-making, and enable more personalized treatment strategies, ultimately helping to reduce errors and improve patient outcomes [163].

Certain complex conditions, such as obesity, highlight the limitations of current, non-AI-based approaches. In the context of obesity, interventions often struggle with personalization because the condition results from a complex interplay of biological, behavioral, and environmental influences, making uniform strategies less effective. Large-scale analyses and meta-reviews have frequently failed to translate scientific findings into practical, individualized strategies. Here, AI-driven systems may offer a way forward by integrating diverse health and behavioral data to deliver adaptive, individualized interventions. Consequently, programs that do not address the specific needs, contexts, and preferences of individuals tend to fall short in achieving lasting, sustainable health improvements [164].

By enabling continuous, multidimensional data capture, AI systems can be designed to address not only the behavioral determinants of obesity but also the underlying pathophysiological processes, such as insulin resistance, dysregulated appetite control, chronic low-grade inflammation, and altered gut–brain axis signaling [165,166,167].

### 6.2. AI-Based Personalized Nutrition

A prime example of AI-enabled personalization in obesity care is personalized nutrition (PN)—a data-driven approach that adapts dietary recommendations to the unique biological, behavioral, and environmental profiles of individuals. Unlike generalized nutritional advice, PN incorporates genetic profiles, metabolic phenotypes, disease risk factors, and lifestyle patterns to create precision-based dietary strategies. Advances in nutrigenomics, which explores gene–nutrient interactions, have provided a strong scientific foundation for these dietary approaches. The integration of AI with nutrigenomics and other multi-omics data has further enhanced the capacity to predict individual dietary responses. ML techniques such as random forests, gradient boosting, multilayer perceptrons, and long short-term memory networks have been applied to forecast postprandial glycemic responses, lipid fluctuations, and weight change trajectories. By enabling precise, individualized dietary strategies, AI-driven PN offers a promising adjunct to obesity management, improving adherence and metabolic outcomes where generic diet plans often fail [168].

Wearable devices and mobile apps now facilitate continuous monitoring of diet, activity, and health metrics, with AI and ML algorithms analyzing these data to produce tailored nutrition recommendations. Emerging systems integrate genetic, metabolic, and lifestyle inputs to design individualized supplements, diet and exercise plans, and microbiome-based nutrient guidance, combining multiple health factors to support comprehensive weight and disease management [169]. Data from such systems can also be integrated into advanced AI architectures, including large language models (LLMs), to further refine personalization and enhance patient engagement.

Recent developments in LLMs enable the translation of complex dietary guidelines into accessible, clinically grounded recommendations for obesity care. An example of this method in practice integrates LLMs with the Retrieval-Augmented Generation framework, ensuring outputs are both personalized and based on validated nutritional data. An AI-driven system using this method was developed to generate customized smoothie recipes for individuals with obesity, targeting calorie control, increased fiber intake, and balanced macronutrient composition [170]. By combining clinical accuracy, real-time personalization, and sustainability principles, this system offers a scalable tool for improving dietary adherence and supporting sustainable weight loss in diverse healthcare settings [170].

Building on this, recent research has evaluated freely accessible models such as ChatGPT in the context of PN for obesity. A comparative analysis using data from the German Food4Me sub-cohort clinical trial evaluated ChatGPT’s dietary recommendations against those generated by the validated Food4Me algorithm. While ChatGPT demonstrated the ability to deliver individualized advice with high accessibility and user engagement, it also produced inconsistent outputs, including inaccuracies in macro- and micronutrient targets relevant to obesity management. These findings highlight both the potential and current limitations of LLM-based dietary counseling, emphasizing the need for refinement, integration with authoritative data sources, and professional oversight before widespread clinical adoption. When optimized for macronutrient balance and fiber intake, such approaches may contribute to improved glycemic regulation and favorable modulation of gut microbiota composition—key factors in the metabolic dysregulation characteristic of obesity [171].

Complementary to such conversational AI approaches, recent work has explored the use of wearable technologies and automated data capture methods to strengthen the accuracy, scalability, and personalization of dietary interventions.

A one-month feasibility study called “AI4Food,” embedded within a prospective, crossover, randomized controlled trial, assessed the application of wearable technologies and mobile platforms in a nutritional weight-loss intervention targeting individuals with overweight and obesity. Ninety-three participants were randomized into two groups: the first used manual data collection methods via validated questionnaires for the initial two weeks, while the second group began with automated data collection through wearable sensors; thereafter, the groups switched methods. Lifestyle, anthropometric, and biological data were collected at each study phase. The intervention achieved a mean weight loss of 2 kg (*p* < 0.001), with significant improvements in BMI, visceral fat, waist circumference, total cholesterol, and HbA1c. Automated monitoring employed the Fitbit Sense smartwatch for physical activity, heart rate, sleep quality, and other physiological parameters; the Freestyle Libre 2 continuous glucose monitoring system, performing measurements every 15 min and read via NFC-enabled smartphones; and a mobile-responsive web platform for real-time food image acquisition and cloud-based storage with precise timestamps. Participants rated the technology favorably (mean System Usability Scale score: 78.27 ± 12.86), and continuous glucose monitoring revealed distinct glycemic response patterns among subgroups. The study produced a substantial dataset and demonstrated that integrating wearable-based automatic data collection into precision nutrition programs is both feasible and well-accepted, offers higher accuracy, and reduces participant burden and continuous monitoring. This approach generates rich datasets for machine learning, providing a solid foundation for implementing and validating AI-driven clinical nutrition tools, while enabling early detection of adverse glycemic patterns and physical inactivity—both of which contribute to insulin resistance, impaired lipid metabolism, and the accumulation of visceral adiposity [172].

### 6.3. Overview of Advanced AI Platforms for Obesity Management

Several advanced AI platforms have emerged that leverage modern computational methods to deliver individualized, evidence-based interventions for obesity management.

The rapid expansion of smartphones and wearable devices has enabled mobile and eHealth applications that facilitate self-monitoring, behavior change, and personalized feedback, often incorporating behavioral models such as the Information–Motivation–Behavioral Skills model to enhance adherence. Evidence from umbrella reviews indicates that mobile app–based and long-term eHealth interventions (≥12 months) can achieve modest but statistically significant reductions in body weight, BMI, and abdominal girth, whereas the effectiveness of stand-alone web-based programs remains inconclusive, particularly without human support. High heterogeneity across studies and limited evaluation in vulnerable populations highlight the need for further research to improve accessibility and integration of these technologies into broader health strategies [167].

One example is a recent single-group pretest–posttest study, conducted on a Southeast Asian cohort, that investigated the feasibility and short-term effectiveness of the Eating Trigger-Response Inhibition Program (eTRIP), a 12-week AI-assisted mobile application developed to support weight management in individuals with overweight and obesity by strengthening self-regulation of eating behaviors. Grounded in a modified temporal self-regulation theory, the program integrates three components: chatbot-based check-ins to identify eating lapse triggers, food image recognition tailored to local dietary patterns, and automated time-based nudges combined with a meal stopwatch. In a 1-week run-in period involving 230 participants, statistically significant improvements were observed in several psycho-behavioral outcomes, including overeating and snacking habits, self-regulation of eating behaviors, depressive symptoms, and physical activity levels, while anxiety remained unchanged. Qualitative feedback indicated that participants valued increased mindfulness in self-monitoring, the personalized nature of reminders and chatbot prompts, the efficiency of image-based food logging, and the simple, appealing user interface. The low attrition rate and high acceptability suggest feasibility for larger-scale evaluation and long-term efficacy studies [173].

Similarly, other AI-driven digital platforms, such as SureMediks, have demonstrated substantial potential in delivering scalable, personalized lifestyle interventions for obesity management. A multinational field trial evaluated SureMediks, an AI-powered digital lifestyle intervention platform combining a mobile application, internet-connected scale, and an expert system to deliver individualized weight-management guidance over 24 weeks. Among 391 participants (58% women) with BMI ranging from 20 to 78 kg/m^2^, all achieved body weight reduction, with a mean loss of 14% (range 4–22%). Nearly all (98.7%) reduced their weight by at least 5%, 75% lost at least 10%, 43% at least 15%, and 9% at least 20%, indicating potential benefits for obesity-related comorbidities. Greater weight loss was associated with female sex, larger accountability circles, and participation in structured challenges, whereas frequent sub-goal reassignment correlated negatively with outcomes. These findings support the role of AI-assisted interventions in producing clinically meaningful weight loss across diverse BMI ranges [174].

In addition to behavioral platforms, AI-based clinical decision support systems are being developed to enhance obesity diagnosis and treatment planning. One such system, designed within the SEMMA data mining framework, integrates predictive modeling with expert-derived treatment recommendations. Using an interactive R Shiny interface, the platform comprises three modules: system introduction, obesity assessment, and results presentation. Five multi-classification algorithms—multinomial logistic regression, decision tree, random forest, support vector machine, and Naive Bayes—were evaluated, with multinomial logistic regression achieving the highest diagnostic accuracy of 97.48% using a reduced feature set. Comparative analysis demonstrated that this performance exceeded or matched other machine learning approaches reported in the literature, while also addressing a critical gap: the integration of actionable treatment guidance. The platform comprises three core modules—system introduction, obesity assessment, and results presentation—and uses expert knowledge with if–then rules to generate personalized interventions. Although the dataset combined real and synthetic data due to class imbalance and relied on BMI as the primary classification criterion, future enhancements are planned to incorporate larger proportions of real longitudinal health data, additional anthropometric indicators (e.g., waist circumference, waist-to-hip ratio, waist-to-height ratio), and advanced models such as gradient boosting and neural networks. By enabling early detection and delivering tailored management strategies, such systems could significantly improve obesity care, particularly in underserved settings [175].

Together, these AI-driven platforms illustrate the growing potential of digital health technologies to deliver effective, scalable, and individualized obesity management.

### 6.4. Limitations and Risks Associated with Artificial Intelligence-Powered Systems

The integration of AI-driven interventions into healthcare offers significant promise; however, it also presents formidable challenges, such as patient privacy, the preservation of human autonomy, and algorithm bias, as well as generalizability issues. Notably, a key consideration involves safeguarding sensitive health data, as AI systems’ reliance on extensive information raises significant risks of breaches and misuse [176].

There is extensive research dedicated to testing and enhancing trust in AI-driven recommendations. One such systematic review, encompassing studies from 2010 to 2023, specifically investigated AI applications in healthcare and their implications for safety, transparency, and ethics. This analysis confirmed that while explainable AI does improve understanding and trust, concerns remain, notably with over 60% of healthcare professionals expressing hesitation due to the nature of AI’s transparency and data insecurity. This gap between patients’ perceptions and privacy and security scenarios in AI-integrated healthcare systems highlights the critical need for transparent communication with patients and the public to demystify AI’s role and foster informed understanding. The urgency of robust cybersecurity was further underscored by the 2024 WotNot data breach. Ultimately, this research concludes that successful AI integration necessitates ethical and technical safeguards, such as bias mitigation, strengthened cybersecurity, and collaborative regulatory guidelines, to responsibly realize AI’s transformative potential in healthcare [176,177].

Addressing these challenges of privacy within healthcare AI necessitates a multifaceted approach to enhance data integrity and security. The inherent reliance of AI systems on extensive datasets intensifies risks such as the reidentification of anonymized data, thereby mandating robust regulatory frameworks and continuous systemic oversight. To mitigate these vulnerabilities, several advanced privacy-preserving technologies (PPTs) are being deployed. Homomorphic Encryption, for instance, facilitates secure computations directly on encrypted sensitive information, proving crucial for applications such as genomic analysis. Concurrently, Differential Privacy ensures individual anonymity through the strategic injection of controlled noise, permitting valuable aggregate data insights without compromising personal identities. Blockchain technology further contributes by offering a decentralized, immutable ledger, significantly enhancing data security, transparency, and traceability for patient records and supply chains. Additionally, the development of synthetic data, exemplified by tools such as Synthea, provides a critical avenue for AI model development and testing without exposing real patient information, despite ongoing complexities in ensuring its realism and comprehensive validation. While these PPTs demonstrate substantial promise in balancing confidentiality with data utility, persistent challenges remain regarding computational efficiency, scalability, and seamless integration with existing healthcare IT infrastructures [176].

Moreover, ensuring AI serves as a supportive tool rather than a replacement for human judgment is crucial to maintaining the vital human element in medical decision-making. Concerns exist that AI may dehumanize medical practice by potentially replacing human professionals and is inherently incapable of expressing empathy, a vital component of holistic patient care [176,178].

Artificial intelligence significantly impacts the foundational doctor–patient relationship, a cornerstone of effective healthcare. The inherent limitations of AI systems in comprehending and reciprocating human emotions, particularly empathy and compassion, directly impede the development of trust and the maintenance of therapeutic connections, thus leading to a more depersonalized and even dehumanized patient experience. Moreover, the integration of AI can introduce a “third party” into clinical encounters, potentially eroding the autonomy of both physicians and patients as they navigate AI-generated recommendations. This dynamic risks undermining shared decision-making, where medical professionals may feel compelled to follow algorithmic advice, thereby questioning their clinical judgment, while patients may perceive AI as the ultimate authority. Furthermore, the opaque “black-box” nature of many AI algorithms, coupled with their training on generalized datasets, can introduce and amplify biases, standardize care in ways that overlook individual nuances, and potentially widen existing health disparities, particularly for minority groups or those with less common conditions. While some argue that AI could offer benefits, such as reducing the impact of shame in specific care contexts or automating routine tasks to free up physician time for patient interaction, these advantages must be carefully weighed against the profound risk of diminishing the irreplaceable human elements of healthcare [179].

Furthermore, the substantial costs associated with AI tools, particularly the enormous computing power and vast datasets required for training and fine-tuning advanced models such as large language models, present a significant financial barrier to entry for many healthcare institutions. These high computational, infrastructural, and operational expenses compound the need for specialized training and robust, high-quality data. Consequently, their accessibility could be severely limited, exacerbating inequities in healthcare resource distribution, as only those systems with considerable budgets can afford the initial investment and ongoing maintenance of such sophisticated AI [178,180].

## 7. Conclusions

Obesity is a chronic and multifactorial condition associated with serious complications such as cardiovascular disease, type 2 diabetes, cancer, liver disease, sleep apnea, bone disorders, and endocrine dysfunctions. Its pathogenesis is shaped by genetic predisposition, epigenetic regulation, alterations of the gut microbiota, circadian misalignment, and the complex activity of gut hormones, including leptin, ghrelin, peptide YY, GLP-1, and cholecystokinin.

Recent progress in research and clinical practice has significantly expanded the possibilities for both treatment and prevention. Lifestyle modification remains the fundamental strategy, but its effectiveness is now reinforced by pharmacological agents that directly influence appetite regulation and energy expenditure. Therapies targeting gut hormones, particularly GLP-1 receptor agonists and related peptide analogs, have demonstrated substantial benefits in reducing body weight and improving glucose homeostasis. Bariatric surgery continues to be the most effective intervention, producing durable weight reduction and favorable hormonal adaptations that enhance metabolic balance and appetite control. At the same time, novel approaches such as gene therapy and the application of artificial intelligence offer the prospect of personalized interventions that may correct underlying metabolic disturbances and optimize clinical outcomes. Preventive actions are equally important in limiting the burden of obesity. Early-life factors, including parental health, maternal smoking, nutritional conditions, and circadian rhythm, play a decisive role in shaping susceptibility to obesity in later years. Addressing these influences through targeted preventive measures can reduce the risk of transmitting vulnerability to future generations.

In conclusion, obesity requires a comprehensive and multidisciplinary strategy that integrates lifestyle changes, pharmacological and hormonal therapies, bariatric surgery, and innovative approaches such as gene therapy and artificial intelligence, alongside preventive measures beginning in early life. Only such an integrated framework can effectively reduce the prevalence of obesity, alleviate its complications, and improve long-term health outcomes.

## Data Availability

No new data were created or analyzed in this study. Data sharing does not apply to this article.

## Abbreviations

The following abbreviations are used in this manuscript:

ACT	Acceptance and Commitment Therapy
AHI	Apnea Hypopnea Index
AI	Artificial intelligence
Akt	Protein kinase B
AMPK	AMP-activated protein kinase
apoB	Apolipoprotein B
Arc	Arcuate nucleus
ATS	Adipocyte-targeting peptide
ATS-9R	9-mer arginine sequence
BAT	Brown adipose tissue
BBB	Blood–brain barrier
BMD	Bone mineral density
BMI	Body mass index
BPA	Bisphenol A
CAAs	Cancer-associated adipocytes
cAMP	Cyclic adenosine monophosphate
CBT	Cognitive Behavioral Therapy
CCK	Cholecystokinin
ChREBP	Carbohydrate-responsive element-binding protein
CRISPRi	CRISPR interference
CVD	Cardiovascular disease
DAG	Diacylglycerol
dCas9	Catalytically inactive Cas9
DM	Diabetes mellitus
EVs	Extracellular vesicles
Fabp4	Fatty acid-binding protein 4
FOXO1	Forkhead box 1
FTO	Fat mass and obesity-associated gene
GLP-1	Glucagon-like peptide-1
GLP1RAs	GLP-1 receptor agonists
GLUT4	Glucose transporter type 4
GSK3	Glycogen synthase kinase-3
HCC	Hepatocellular carcinoma
HDL	High-density lipoprotein
HDL-C	High-density lipoprotein cholesterol
HF	Heart failure
HRpEF	Heart failure with preserved ejection fraction
IL	Interleukin
IR	Insulin resistance
IRS-1	Insulin receptor substrate 1
IRS-2	Insulin receptor substrate 2
LDL	Low-density lipoprotein
LEP	Leptin
LEPR	Leptin receptor gene
LIR	Liraglutide
LLMs	Large language models
LV	Left ventricle
MASLD	Metabolic Dysfunction-Associated Steatotic Liver Disease
MBSR	Mindfulness-Based Stress Reduction
MC4R	Melanocortin 4 receptor
ML	Machine learning
mTORC1	Mammalian target of rapamycin complex 1
OSA	Obstructive sleep apnea
PDE3B	Phosphodiesterase 3B
PI3K	Phosphoinositide 3-kinase
PKA	Protein kinase A
PN	Personalized nutrition
PP1	Protein phosphatase 1
PP2A	Protein phosphatase 2A
PPAR-γ	Peroxisome proliferator-activated receptor gamma
PPTs	Privacy-preserving technologies
PRS	Polygenic risk score
PTP1B	Protein tyrosine phosphatase 1B
PYY	Peptide YY
RAAS	Renin–angiotensin–aldosterone system
RYGB	Roux-en-Y gastric bypass
SCFAs	Short-chain fatty acids
SG	Sleeve gastrectomy
SOCS3	Suppressor of cytokine signaling 3
SREBP-1c	Sterol regulatory element-binding protein 1
TALENs	Transcription activator-like effector nucleases
T2DM	Type 2 diabetes mellitus
TNF-α	Tumor necrosis factor alpha
VAT	Visceral adipose tissue
WAT	White adipose tissue
